# Plasma lipids connecting olfaction with cognition and physical function

**DOI:** 10.1038/s41598-026-43857-2

**Published:** 2026-03-10

**Authors:** Erin E. Greig, Susan M. Resnick, Luigi Ferrucci, Qu Tian

**Affiliations:** 1 Longitudinal Studies Section, Translational Gerontology Branch, National Institute on Aging, 251 Bayview Blvd., Suite 100, Baltimore, MD 21224 USA; 2 Laboratory of Behavioral Neuroscience, National Institute on Aging, 251 Bayview Blvd., Suite 100, Baltimore, MD 21224 USA

**Keywords:** Olfaction, Lipids, Cognition, Physical function, Aging, Alzheimer’s disease, Biochemistry, Neuroscience

## Abstract

Olfactory deficit is an early sign of neurodegenerative diseases, such as Alzheimer’s disease (AD), and is related to age-related cognitive and physical function decline. However, underlying biological processes are not fully understood. Identifying shared lipid metabolites may shed some light. In 656 Baltimore Longitudinal Study of Aging participants (mean age:70.5 years), we examined associations of plasma lipids with olfaction, cognitive impairment (including AD), cognition, and physical function, after adjusting for demographics. Plasma lipid metabolites, assayed via FIA- and LC-mass spectrometry, were analyzed as 6 lipid classes. Odor identification was measured via the 16-item Sniffin’ Sticks. Cognition was measured via a neuropsychological battery. Physical function measures included gait speed, chair stands, and balance. Very long-chain and long-chain sphingomyelins and glycosylceramides were positively associated with olfaction, select cognitive measures (Trail Making Test-Part A, Digit Symbol Substitution Test, Purdue Pegboard Test), and physical function. Sphingomyelins were also associated with memory (California Verbal Learning Test immediate recall) and cognitive impairment (all *p* < 0.05). Only very long-chain sphingomyelins and glycosylceramides affected associations between olfaction and outcome measures (Δβ:10-25.9%). Plasma sphingomyelins and glycosylceramides, especially those with very long-chain carbons, may at least in part explain the relationship between olfaction and functional outcomes. Future omics studies may provide additional mechanistic insights.

**Supplementary Information:**

The online version contains supplementary material available at 10.1038/s41598-026-43857-2.

## Introduction

The sense of smell, or olfactory function, is essential in our daily lives, including the ability to taste food and detect hazards^1^. Olfactory deficits are recognized as early signs of neurodegenerative diseases and age-related cognitive and physical function decline^2–5^, but the underlying mechanisms are not fully understood. The olfactory system detects the odorant molecules in the peripheral olfactory system, which is transduced to the central olfactory system, including the olfactory bulb and other related brain areas, such as the olfactory cortex, orbitofrontal cortex, and limbic system^6,7^. While brain areas related to olfaction may explain the associations between olfaction and cognitive and physical function, other contributing factors and mechanisms have not been investigated^2,8^.

Searching for biomarkers associated with olfaction may shed light on why olfaction serves as a marker of future cognitive impairment and declines in cognitive function and physical function. Certain lipid species in the nasal mucosa are correlated with olfactory deficit, suggesting the contribution of lipids to olfactory function^9,10^. The olfactory mucosa, a key tissue localized in the nasal cavity, is crucial in detecting odorants and transmitting olfactory signals to the brain^11^. The olfactory epithelium, a major component of the olfactory mucosa, is home to the sensory terminations of olfactory neurons that bring the olfactory signal to the olfactory bulb, where their axons synapse with other neurons that project information to specific brain areas^11^. The plasma membrane of olfactory sensory neurons contains critical components of signaling proteins and key lipids, which enable signal transduction to the brain^12,13^. The metabolic properties and functions of the nasal mucosa and olfactory bulb share similarities with those of the limbic system neurons. There is a two-way modulation between olfactory perception and hypothalamic neurons in maintaining metabolic homeostasis, covering the nasal mucosa, the olfactory bulb, and the limbic system^14–16^. Lipids in circulation are also associated with cognitive impairment, cognition, and physical function^17^. In addition, circulating lipids are associated with brain white matter health, diet, and body fat, which may explain the associations between olfaction and cognitive and physical function^18–20^.

Because the function of lipids depends on their biochemical structure, in this study, we analyzed lipid classes based on the length of the carbon chain^21,22^. Specifically, we aim to identify plasma lipids related to both olfaction and outcome measures of cognitive impairment, cognition, and physical function. For shared lipids associated with both olfaction and cognitive and physical function, we further examined whether they would affect the relationships between olfaction and cognitive and physical function.

## Methods

### Study population

Participants were from the Baltimore Longitudinal Study of Aging (BLSA), an observational study with continuous enrollment of community-dwelling adult volunteers since 1958^23,24^. The BLSA enrolls participants who do not have any major chronic illnesses or cognitive impairment. These exclusion criteria only apply at study enrollment. Once enrolled, participants return to the National Institute on Aging Clinical Research Unit for follow-up every 4 years before the age of 60, every 2 years for those aged 60–79, and every year for those aged 80 and older. We identified 656 participants (55% women, 30% Black) who had concurrent measures of olfaction, plasma metabolomics, and cognitive and physical function at their most recent visit in the BLSA. Data were collected between 2015 and 2020. At all visits, BLSA participants provided informed written consent. The Institutional Review Board of the National Institutes of Health approved the BLSA study protocol. All research was performed in accordance with relevant guidelines/regulations and in accordance with the Declaration of Helsinki.

### Olfactory function

Olfaction was assessed using the odor identification 16-item Sniffin’ Sticks test^25^. After smelling each stick, participants were instructed to select only one from 4 odorant choices. The correct choice was scored as one. The test score ranged between 1 and 16. A higher score indicates higher olfactory function in odor identification. Two test versions of the Sniffin’ Sticks were alternated, with the initial version randomized at baseline to minimize learning effects.

### Cognitive function and diagnoses of cognitive impairment and dementia

Cognitive measures included global mental status, verbal memory, executive function, attention, processing speed, and manual dexterity. Global mental status was measured using the Mini-Mental State Examination (MMSE)^26^. Psychomotor speed was measured using the Digit Symbol Substitution Test (DSST)^27^. Attention was measured using the Trail Making Test part A (TMT-A). Executive function was measured using TMT part B (TMT-B)^28^. Manual dexterity was measured using the Purdue Pegboard Test^29^. Verbal memory was assessed via the California Verbal Learning Test (CVLT) immediate recall score^30^. Diagnoses of Dementia, Alzheimer’s disease, and mild cognitive impairment were determined using the Diagnostic and Statistical Manual of Mental Disorders, Third Edition, Revised (DSM-III-R)^31^, the National Institute of Neurological and Communicative Disorders and Stroke-Alzheimer’s Disease and Related Disorders Association (NINCDS-ADRDA) criteria^32^ and the Petersen criteria^33^, respectively. The cognitive status of participants was adjudicated at a research diagnostic case conference if they had a Blessed Information-Memory-Concentration Test score ≥ 4, Clinical Dementia Rating (CDR) score ≥ 0.5, or if there were concerns about cognitive status^34,35^.

### Physical function outcomes

Physical function measures included 6-meter usual gait speed, 400-meter walk time, and physical function via the Health, Aging and Body Composition Physical Performance Battery (HABCPPB). HABCPPB included 6-meter usual and narrow walking speed, chair stands, and balance^36^. Participants with a usual gait speed slower than 1.0 m/s were considered as having physical function impairment.

### Plasma lipids

Blood was collected from the antecubital vein of participants, between 07:00 and 08:00, after at least 8 h of overnight fasting and abstention from smoking and medications^37^. Participants were also instructed to abstain from exercise during the fasting period. Following the guidelines for biomarker studies, blood samples were stored at −4 °C. Within 4 h of collection, blood samples were centrifuged at an RCF of 1229 x g for 15 min (Beckman Coulter Allegra X-14R Refrigerated Centrifuge with SX4750A rotor), aliquoted, and stored at −80 °C^38^.

Lipid metabolites in the plasma were measured using the MxP Quant 500 kit (Biocrates Life Sciences AG, Innsbruck, Austria), which assesses up to 638 metabolites. The MxP Quant 500 kit followed a 5500 QTRAP mass spectrometer protocol (Sciex, Framingham, MA, USA). Flow injection analysis tandem mass spectrometry (FIA-MS/MS) was used to quantify plasma lipids, including acylcarnitines, lysophosphatidylcholines, phosphatidylcholines, ceramides, sphingomyelins, cholesteryl esters, and triglycerides. Liquid chromatography with tandem mass spectrometry (LC–MS/MS) was used to quantify plasma fatty acids. Metabolites exceeding 30% of values below the limit of detection were excluded from the analysis. For the remaining metabolites, the missing values were imputed as half of the minimum value^39^. Lipid metabolites were first grouped by the biochemical nature of the molecules, including acylcarnitines, ceramides, glycosylceramides, glycerolipids and cholesterol esters, sphingomyelins, and triglycerides^21^. Within each biochemical class, lipids were further categorized based on carbon chain length. Lipids with less than 6 carbons were grouped as short-chain, 6 to10 carbons as medium-chain, 14 to 20 carbons as long-chain, and 22 carbons or more as very long-chain. Using a consistent approach, we computed the sum of log2-transformed values as composite scores for lipid class variables^21^. The beta-oxidation rate was calculated as the ratio of the sum of acetylcarnitine (C2) and propionyl carnitine (C3) over carnitine (C0)^40–42^.

### Potential contributing factors of white matter integrity, diet, and visceral fat

To further understand potential mechanisms, we explored factors that may be associated with circulating lipids, including brain white matter health, diet, and visceral fat in subsamples with available data. White matter integrity was measured using fractional anisotropy (FA) via Diffusion Tensor Imaging at 3T. FA describes the directionality of water molecules within white matter tracts. A higher FA value indicates higher white matter integrity. Details of brain DTI processing are described elsewhere^43^. Diet was assessed using a food frequency questionnaire as described previously^44^. Diet measures included protein consumption, fat consumption, the Alternative Healthy Eating Index, and the Mediterranean-like diet score. Visceral fat was assessed by computed tomography (CT) scan^45^.

### Statistical analysis

We first performed logistic regression to confirm the association between olfaction and cognitive impairment. We then performed linear regression to confirm the associations between olfaction and cognitive and physical function. To identify shared lipid markers, we examined the associations of each lipid class with olfaction and cognitive and physical function using multivariable linear regression. Because of known associations of age, sex, and race with olfaction, cognition, and physical function, all models were adjusted for age, sex, and race^46–52^. Models were also adjusted for two versions of the odor identification test. Because body height was correlated with gait speed, we additionally adjusted for height in models of physical function measures. Because educational attainment was related to cognition, we additionally adjusted for education in models of cognitive outcomes.

To determine whether lipid markers may affect the associations between olfaction and cognitive and physical function measures, we compared models with and without the shared lipids. As a supplementary analysis, we tested whether lipid markers (i.e., mediators) would mediate the association between olfaction (i.e., exposure) and functional outcomes. Quasi-Bayesian resampling simulations with 500 simulations per model were conducted to assess the uncertainty of effects in parametric linear regression models.

In the secondary, exploratory analyses, we examined associations between lipid markers with white matter integrity, diet, and visceral fat using multivariable linear regression, after adjusting for demographics. In this exploratory study, statistical significance was set at two-tailed *p* < 0.05. We also highlighted associations that passed multiple testing corrections using False Discovery Rate (FDR). We used R Studio version 4.2.1 (Boston, MA) for all analyses.

## Results

The characteristics of the participants are shown in Table 1. The mean age was 70.5 years. 55% were women. 30% were Black. 3% had cognitive impairment, and 23% had slow gait at the time of assessment (Table 1).

**Table 1:** Participants’ characteristics.

	Overall sample (*n* = 656)
	Mean (SD) [range] or *N* (%)
Demographics	
Age, years	70.5 (4.7) [22.4–100.8.4.8]
Women	364 (55%)
Black	199 (30%)
4-year college or above	563 (86%)
Height, cm	167.4 (9.3) [143.8–196.2.8.2]
Visceral fat, cm^2^	111.7 (72.6) [6.3–352.8.3.8] (*n* = 570)
Apolipoprotein E epsilon 4 carriers	159 (24.2%)
Disease conditions	
Vascular diseases	353 (53.8%)
High blood pressure or hypertension	304 (46.3%)
Peripheral neuropathy	65 (9.9%)
Heart attack or myocardial infarction	22 (3.4%)
Transient ischemic attack	20 (3.0%)
Stroke	18 (2.7%)
Coronary artery disease	16 (2.4%)
Heart failure or congestive heart failure	14 (2.1%)
High cholesterol triglycerides	362 (55.2%)
Diabetes	100 (15.2%)
Stomach gastric or duodenal ulcer	49 (7.5%)
Chronic bronchitis, emphysema, or chronic obstructive pulmonary disease	21 (3.2%)
Parkinson’s disease	1 (0.2%)
Odor identification scores	10.8 (2.7) [1–16]
Cognitive impairment or dementia	17 (3%)
Cognitive function	
Mini-Mental State Examination	28.6 (1.4) [22–30] (*n* = 642)
Digit Symbol Substitution Test	43.0 (12.5) [10–84] (*n* = 631)
Trail Making Test Part A, seconds	32.2 (12.0) [11–119] (*n* = 647)
Trail Making Test Part B, seconds	80.6 (42.1) [26–300] (*n* = 631)
Purdue Pegboard dominant hand performance	12.6 (2.2) [5.5–19.0] (*n* = 638)
Purdue Pegboard non-dominant hand performance	12.2 (2.2) [3.5–18.0] (*n* = 641)
California Verbal Learning Test, immediate recall	52.9 (11.8) [20–80] (*n* = 643)
Physical impairment (usual gait speed < 1.0 m/sec)	151 (23%)
Physical function	
Usual 6-meter gait speed, m/s	1.14 (0.23) [0.36–1.88] (*n* = 644)
400-meter walk time, seconds	284.2 (63.2) [181.1–600.3.1.3] (*n* = 609)
Health, Aging and Body Composition Physical Performance Battery	2.56 (0.59) [0.32–3.83] (*n* = 642)

### Associations of lipids with olfaction and cognition

We found that olfaction was associated with cognitive impairment and cognitive performance, consistent with the previous literature^53^. Specifically, each unit higher in odor identification scores was associated with 17.4% lower odds of being cognitively impaired (odds ratio = 0.826, *p* = 0.047), and associated with higher cognitive performance measured by MMSE, CVLT immediate recall, TMT-A, DSST, and the pegboard test (all *p* < 0.05) (Supplementary Table 1). The association between odor identification scores and executive function by TMT-B was not significant (*p* > 0.05) (Supplementary Table 1).

**Fig. 1:**
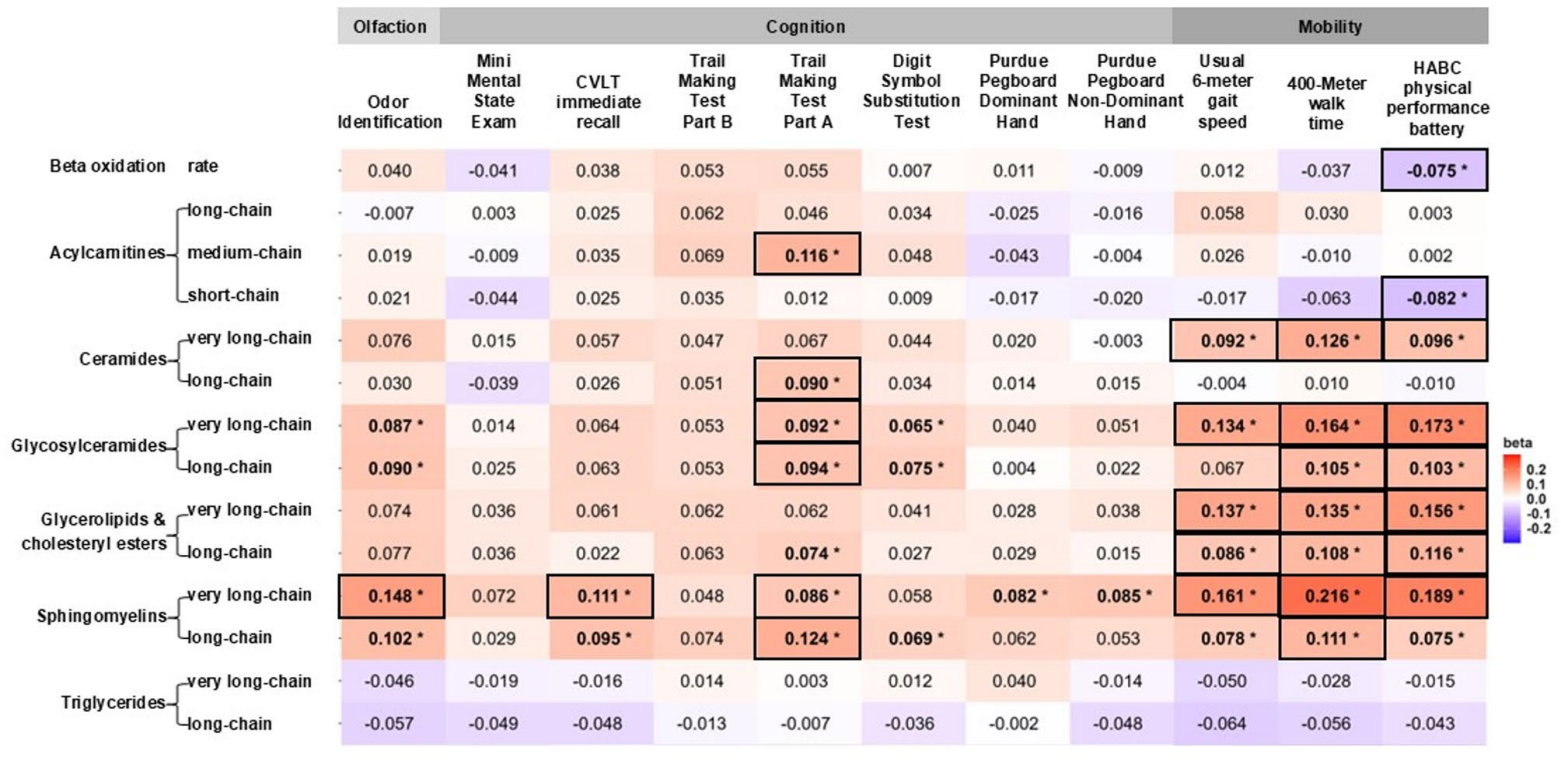
Heatmap of the associations of lipids with olfaction and cognitive and physical performance. Legend: Values of olfaction, cognition, and physical function measures are standardized to Z-scores. Z scores of TMT-A, TMT-B, and 400-meter walk time were flipped to be consistent with the directionality of other outcome measures. * indicates significance at *p* < 0.05. Outlined boxes indicate associations at pFDR < 0.05. CVLT: California Verbal Learning Test, HABC: Health, Aging and Body Composition.

**Fig. 2:**
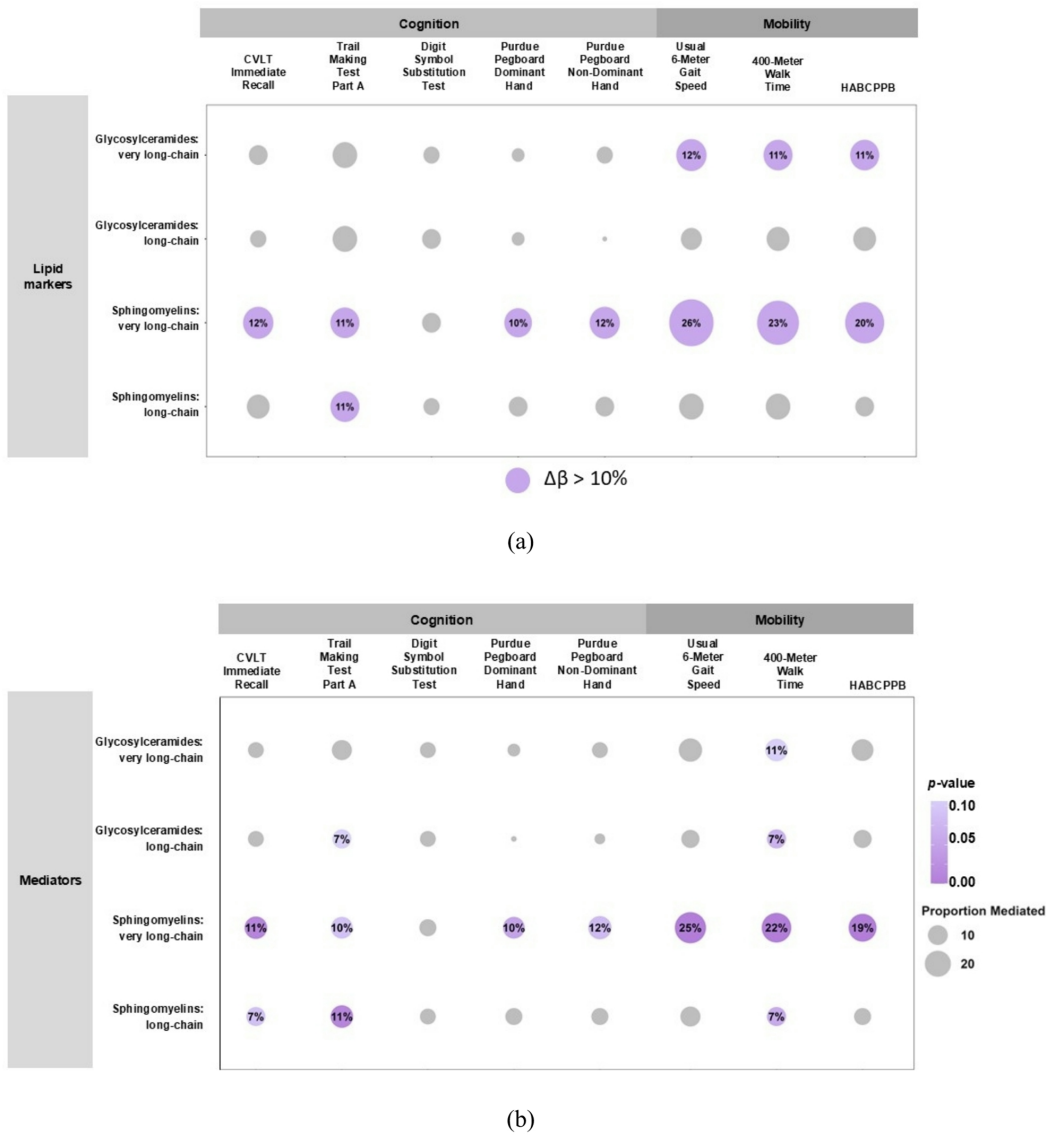
The contribution of lipid markers to the associations between olfaction and cognitive and physical function outcomes. **a** Circles in color indicate lipids that attenuate the regression coefficient of olfaction in association with cognitive and physical function outcomes by more than 10%. **b** Circles filled in color indicate significant mediation effects.

Olfaction was associated with select lipid variables, including glycosylceramides (very long-chain and long-chain) and sphingomyelins (very long-chain and long-chain) (β: 0.072, 0.090, 0.127, 0.098, respectively, all *p* < 0.05) (Table 2; Fig. 1, Supplementary Table 2). There were no significant associations between olfaction and other lipids, including beta oxidation, acylcarnitines, ceramides, glycerolipids and cholesteryl esters, and triglycerides. Sphingomyelins and glycosylceramides were also associated with cognitive measures, including TMT-A, DSST, pegboard performance, and CVLT. Other lipid measures of acylcarnitines, ceramides, and glycerolipids were associated with TMT-A (Table 2; Fig. 1, Supplementary Table 2). Very long-chain sphingomyelins slightly attenuated the associations of olfaction with cognitive measures of CVLT, TMT-A, and Pegboard performance (Δβ: 10.1–12.3%; Table 3; Fig. 2a). Long-chain sphingomyelins slightly attenuated the association of olfaction with TMT-A (Δβ: 11.3%; Table 3; Fig. 2a). Additional mediation analyses with bootstrapping revealed that lipids that affected the associations by at least 10% also showed significant mediation effects or a trend (*p* < 0.05 or 0.05 < *p* < 0.10, Fig. 2b). Glycosylceramides did not substantially affect the association between olfaction and cognitive measures (Δβ < 10%; Table 3; Fig. 2a).

**Table 2:** Associations of lipids with olfaction and cognitive and physical impairment.

Lipids	Olfaction(Odor identification scores)		Cognitive impairment		Physical impairment	
	Beta (95% CI)	p-value	Odds ratio (95% CI)	p-value	Odds ratio (95% CI)	p-value
Beta oxidation rate	0.033 (-0.037, 0.102)	0.354	0.942 (0.573, 1.608)	0.819	1.136 (0.920, 1.407)	0.239
Acylcarnitines: long-chain	-0.005 (-0.074, 0.063)	0.879	1.209 (0.696, 2.312)	0.531	1.129 (0.916, 1.403)	0.263
Acylcarnitines: medium-chain	0.016 (-0.054, 0.085)	0.654	1.025 (0.565, 2.111)	0.942	1.016 (0.818, 1.273)	0.889
Acylcarnitines: short-chain	0.017 (-0.053, 0.087)	0.628	0.913 (0.559, 1.541)	0.723	1.198 (0.971, 1.487)	0.096
Ceramides: very long-chain	0.069 (-0.004, 0.142)	0.065	1.228 (0.708, 2.162)	0.468	0.896 (0.719, 1.116)	0.326
Ceramides: long-chain	0.025 (-0.046, 0.097)	0.485	0.858 (0.498, 1.439)	0.570	1.210 (0.977, 1.506)	0.085
Glycosylceramides: very long-chain	0.072 (0.002, 0.142)	0.044	0.955 (0.568, 1.603)	0.86	0.792 (0.643, 0.974)	0.028
Glycosylceramides: long-chain	0.077 (0.006, 0.148)	0.033	0.608 (0.346, 1.048)	0.077	0.915 (0.742, 1.130)	0.409
Glycerolipids & cholesteryl esters: very long-chain	0.061 (-0.009, 0.131)	0.087	0.839 (0.487, 1.434)	0.521	0.920 (0.747, 1.134)	0.434
Glycerolipids & cholesteryl esters: long-chain	0.070 (-0.003, 0.143)	0.061	0.866 (0.471, 1.563)	0.638	1.015 (0.811, 1.273)	0.895
Sphingomyelins: very long-chain	0.127 (0.056, 0.198)	<0.001	0.567 (0.328, 0.964)	0.037	0.714 (0.571, 0.887)	0.003
Sphingomyelins: long-chain	0.098 (0.023, 0.174)	0.011	0.592 (0.324, 1.054)	0.079	0.900 (0.716, 1.130)	0.363
Triglycerides: very long-chain	-0.036 (-0.104, 0.032)	0.302	1.373 (0.806, 2.368)	0.246	1.283 (1.039, 1.590)	0.021
Triglycerides: long-chain	-0.046 (-0.115, 0.023)	0.195	1.146 (0.652, 2.025)	0.635	1.314 (1.059, 1.635)	0.013

**Table 3:** Lipid markers on the associations between olfaction and cognitive and physical function outcomes.

	Model 1		Model 1 + glycosylceramides						Model 1 + sphingomyelins					
			Very long-chain			Long-chain			Very long-chain			Long-chain		
	β (95% CI)	p-value	β (95% CI)	p-value	Δβ	β (95% CI)	p-value	Δβ	β (95% CI)	p-value	Δβ	β (95% CI)	p-value	Δβ
CVLT	0.532 (0.191, 0.874)	0.002	0.508 (0.165, 0.850)	0.004	4.6%	0.509 (0.167, 0.852)	0.004	4.3%	0.469 (0.126, 0.812)	0.007	11.9%	0.494 (0.151, 0.836)	0.005	7.2%
TMT-A	-0.464 (-0.808, -0.119)	0.008	-0.426 (-0.771, -0.081)	0.016	8.2%	-0.427 (-0.771, -0.082)	0.015	8.0%	-0.413 (-0.760, -0.066)	0.02	11.0%	-0.411 (-0.756, -0.067)	0.019	11.3%
DSST	0.594 (0.292, 0.896)	0.0001	0.569 (0.265, 0.869)	0.0002	4.3%	0.567 (0.292, 0.896)	0.0001	4.7%	0.564 (0.266, 0.871)	0.0002	5.2%	0.569 (0.267, 0.871)	0.0002	4.4%
Pegboard dominant	0.09 (0.035, 0.146)	0.001	0.088 (0.032, 0.143)	0.002	3.0%	0.091 (0.035, 0.146)	0.001	-0.3%	0.081 (0.026, 0.137)	0.004	10.1%	0.086 (0.030, 0.141)	0.002	5.1%
Pegboard nondominant	0.078 (0.023, 0.133)	0.006	0.074 (0.019, 0.130)	0.009	4.5%	0.077 (0.021, 0.132)	0.007	1.6%	0.068 (0.013, 0.124)	0.016	12.3%	0.074 (0.018, 0.130)	0.009	5.0%
Usual gait speed	0.008 (0.001, 0.014)	0.020	0.007 (0.0003, 0.013)	0.041	12.5%	0.007 (0.0007, 0.014)	0.03	6.3%	0.006 (-0.0008, 0.012)	0.084	25.9%	0.007 (0.0006, 0.014)	0.033	8.1%
400m time	-2.86 (-4.64, -1.08)	0.002	-2.55 (-4.31, -0.79)	0.005	11.0%	-2.65 (-4.43, -0.87)	0.004	7.4%	-2.21 (-3.96, -0.46)	0.013	22.8%	-2.63 (-4.41, -0.84)	0.004	8.2%
HABCPPB	0.027 (0.011, 0.043)	0.0008	0.024 (0.009, 0.040)	0.002	10.7%	0.025 (0.010, 0.041)	0.002	6.7%	0.022 (0.006, 0.037)	0.007	20.3%	0.026 (0.010, 0.042)	0.001	4.9%

### Associations of lipids with olfaction and physical function

We confirmed that olfaction was associated with physical function, including 6-meter usual gait speed, 400-meter walk time, and HABCPPB scores (all *p* < 0.05) (Supplementary Table 1). Lipid classes of ceramides, glycosylceramides, glycerolipids, and sphingomyelins were associated with gait speed, 400 m walk time, and HABCPPB scores. Beta-oxidation rate and short-chain acylcarnitines were also associated with HABCPPB scores (Fig. 1, Supplementary Table 2). Very long-chain sphingomyelins and glycosylceramides attenuated the associations of olfaction with physical function measures to some extent (Δβ: 20.3–25.9%; and β: 10.7–12.4%, respectively; Table 3; Fig. 2a). Additional mediation analyses with bootstrapping revealed that lipids that affected the associations by at least 10% also showed significant mediation effects or a trend (*p* < 0.05 or 0.05 < *p* < 0.10, Fig. 2b). Long-chain glycosylceramides and sphingomyelins did not substantially affect the association between olfaction and cognitive measures (Δβ < 10%; Table 3; Fig. 2a).

### Potential contributing factors of the brain white matter, diet, and visceral fat

Identified glycosylceramides (very long-chain and long-chain) and sphingomyelins (very long-chain and long-chain) were associated with higher white matter integrity localized to the corpus callosum and temporal areas. Specifically, higher levels of sphingomyelins and glycosylceramides were associated with higher white matter fractional anisotropy (FA) in select tracts, including the hippocampal part of the cingulum and fornix (Supplementary Table 3). Higher levels of sphingomyelins were associated with higher FA in the corpus callosum. Higher long-chain glycosylceramides were associated with higher FA in the uncinate fasciculus. Higher glycosylceramides were associated with higher Mediterranean-like diet scores. Higher sphingomyelins were associated with more animal protein intake and less vegetable protein intake (Supplementary Table 3). Both sphingomyelins and glycosylceramides were negatively associated with visceral fat (Supplementary Table 3).

## Discussion

Among community-dwelling adults, sphingomyelins and glycosylceramides with long-chain and very long-chain carbons are associated with both olfaction and cognitive and physical function outcomes. These lipids, particularly very long-chain, attenuate the associations between olfaction and cognitive and physical function outcomes to some extent. The supplementary mediation analyses support that these lipids also show significant mediation effects. These circulating lipids may indicate underlying mechanisms that warrant further investigation^9,10^.

Of several lipid classes examined, only sphingomyelins and glycosylceramides are linked to olfaction. These two sphingolipid classes are structurally related to ceramides via the sphingomyelin cycle^54^. The sphingomyelin cycle produces lipid metabolites with different chain lengths, which can change the functional properties of sphingomyelins and glycosylceramides. Synthesis of very long-chain sphingomyelins is regulated by cholesterol homeostasis^55^. Both lipids are large constituents of plasma membranes, localized to membrane rafts, and involved in signal transduction pathways^56^. The connection between these lipids and olfaction may be due to the important roles of these lipids in plasma membranes and signal transduction pathways. There is evidence that sphingomyelins and glycosylceramides are important and detectable lipid components in the nasal and sinus mucosa^9,10^. In aging and neurodegeneration, the sphingomyelin cycle modifies the lipid raft composition of the olfactory epithelium, which may lead to impaired signal transduction and olfactory deficit^56,57^.

Sphingomyelins and glycosylceramides are also associated with cognition and physical function, consistent with previous reports that lower plasma and brain lipid raft sphingomyelin concentrations are associated with preserved cognitive and physical functions^17,58–61^. Impaired lipid metabolism, especially sphingolipids, assessed in circulation, is not only associated with declines in cognition and mobility^17,62,63^ but also linked to neurodegenerative diseases and clinical progression^64–68^. Specifically, impaired sphingolipid metabolism is related to amyloid and tau aggregation in Alzheimer’s disease^64,65,68,69^, alpha-synuclein aggregation and dopaminergic neuron loss in Parkinson’s disease^64,65,67^, and disrupted myelination processes in multiple sclerosis^64,66^. Glycosylceramides have been implicated in Parkinson’s disease (PD), though a detailed understanding of the impact on plasma concentrations of glycosylceramides in this disease and other neurodegenerative diseases remains unknown^69,70^. Notably, the carbon chain length can determine the properties of lipids, including complex-forming affinities, intracellular transport pathways, and lipid raft interactions and functionality^71^. In addition to sphingomyelins and glycosylceramides, beta oxidation rate, acylcarnitines, ceramides, and glycerolipids and cholesteryl esters are also related to specific cognitive and physical function measures. Consistent with previous findings, higher levels of ceramides, glycerolipids, and cholesteryl esters are linked to higher cognitive and physical function, such as verbal memory, manual dexterity, and gait speed^17,43^. Higher short-chain acylcarnitines and higher beta oxidation rate are linked to lower physical function. Acylcarnitines are released into circulation with dysfunctional fatty acid (beta) oxidation and may act as markers of dysfunctional mitochondria, which may lead to poor cognitive and physical function^72,73^. These findings are in line with previous studies that higher levels of acylcarnitines are associated with a greater decline in grip strength and lower cognitive function^21,74^.

Very long-chain and long-chain sphingomyelins and glycosylceramides are shared lipids with olfaction and cognitive and physical function outcomes. Only very long-chain lipids affect the associations between olfaction and functional outcomes, and their effects appear stronger for physical function than cognition. Very long-chain sphingomyelins attenuate the associations of olfaction with cognitive and physical function outcomes, while very long-chain glycosylceramides attenuate the association with physical function only. Impaired lipid metabolism appears the common contributor to the deficits in olfaction and cognition, and neurodegeneration and may trigger common underlying pathways, such as damage in membrane structure, inflammation or neuroinflammation, and reduced energy metabolism and neuronal function. Plasma lipids may reflect the lipid composition in the nasal mucosa, which determines the stability and integrity of olfactory-related membranes and related signal transduction^9^. Plasma lipids may also be related to brain membrane composition, such as myelination^56^, which affects olfaction, cognition, and physical function. In fact, both sphingomyelins and glycosylceramides are involved in the structural stability of myelin^56,75,76^. Alterations in these lipids and a dysfunctional sphingomyelin cycle due to aging or neurodegeneration may lead to compositional changes in white matter structure important for signal transduction and energetics, and affect brain areas responsible for olfaction, cognition, and physical function. Our exploratory analyses show that identified lipids are linked to white matter integrity, particularly in tracts important for movement coordination and memory, such as the corpus callosum, the hippocampal part of the cingulum, and the fornix. These DTI findings may suggest that plasma lipids likely mediate physical function more substantially than cognition via myelin-dependent motor coordination. Whether white matter integrity mediates the association between olfaction and functional outcomes requires further investigation. Diet may be another contributing factor which can lead to inflammation, although we only observed a few associations of plasma lipids with Mediterranean-like diet score and animal and vegetable protein intake. Visceral fat is expectedly associated with identified lipids but further adjustment for visceral fat did not substantially affect the associations between olfaction and functional outcomes (data now shown). Future studies are warranted to investigate biological substrates or processes underlying these lipids and functional outcomes.

This study has notable strengths. First, the investigation of multiple lipid species identifies the specificity of the lipid associations with olfaction. Second, the quantification of lipid species based on the chain length provides additional insights into the importance of the chemical structure of lipids. Third, a diverse array of cognitive and physical function outcomes allows us to examine various domains in cognition and physical function. This study also has limitations. First, this study is limited to an exploratory cross-sectional analysis, which does not establish temporality or address causation. Future longitudinal studies are warranted to validate these findings. Second, the study population tends to be healthier than the general population and has a relatively low prevalence of cognitive impairment or dementia. Third, cardiometabolic conditions or lifestyle factors may have potential confounding effects, which warrant further investigation.

In conclusion, sphingomyelins and glycosylceramides are linked to both olfaction and cognitive and physical function outcomes. Very long-chain lipids may at least in part explain the relationship between olfaction and cognitive and physical function. Future studies are warranted to investigate underlying biological substrates or processes. Studies involving omics biomarkers, such as the microbiome, may provide additional mechanistic insight into these relationships.

## Supplementary Information

Below is the link to the electronic supplementary material.

Supplementary Material 1

## Data Availability

Data are available upon request via the BLSA website portal (https://www.blsa.nih.gov/how-apply). All requests are reviewed by the BLSA Data Sharing Proposal Review Committee (Principal Investigator: Dr. Luigi Ferrucci, National Institute on Aging, NIH) and are subject to approval from the National Institutes of Health Institutional Review Board.
