# Supplementary material for: Plasma lipids connecting olfaction with cognition and physical function
Source: Sci Rep. 2026 Mar 10;16:15168. doi: 10.1038/s41598-026-43857-2 (PMC13171867; doi:10.1038/s41598-026-43857-2)
Supplement: Supplementary file 1 — Supplementary Material 1 [file 41598_2026_43857_MOESM1_ESM.docx]

**Supplementary Table 1. Associations between olfaction and cognitive and physical function outcomes**

| **Cognitive impairment (including AD dementia)** | **Odds ratio (95% CI), p-value** |
| --- | --- |
|  | 0.826 (0.680, 0.997) 0.047 |
| **Cognitive function** | **beta (95% CI) p-value** |
| Mini-Mental State Examination | 0.067 (0.037, 0.097) <0.001 |
| CVLT immediate recall | 0.045 (0.016, 0.074) 0.002 |
| Trail-Making Test part B | -0.017 (-0.046, 0.013) 0.272 |
| Trail-Making Test part A | -0.039 (-0.067, -0.010) 0.008 |
| Digit Symbol Substitution Test | 0.048 (0.023, 0.072) <0.001 |
| Purdue Pegboard dominant | 0.040 (0.016, 0.065) 0.001 |
| Purdue Pegboard non-dominant | 0.035 (0.010, 0.060) 0.006 |
| **Slow gait** | **Odds ratio (95% CI) p-value** |
|  | 0.942 (0.865, 1.024) 0.160 |
| **Physical function** | **Beta (95% CI) p-value** |
| Usual 6-meter gait speed | 0.033 (0.005, 0.061) 0.020 |
| 400-meter walk time | -0.045 (-0.074, -0.017) 0.002 |
| HABCPPB | 0.046 (0.019, 0.073) 0.001 |

Note: Values of cognition and mobility measures are standardized to Z-scores. All models were adjusted for age, sex, and race and additionally adjusted for education and height for cognition and mobility outcomes, respectively. CVLT: California Verbal Learning Test, HABCPPB: Health Ageing and Body Composition Physical Performance Battery.

**Supplementary Table 2. Associations between lipids and cognitive and physical function measures**

|  | **Cognition** | | | | | | **Mobility** | | | | | |
| --- | --- | --- | --- | --- | --- | --- | --- | --- | --- | --- | --- | --- |
|  | **Mini-Mental State Exam** | **CVLT** | **Trail Making Test Part B** | | **Trail Making Test Part A** | **Digit Symbol Substitution Test** | **Pegboard Dominant** | **Pegboard non-dominant** | **Usual 6-m gait speed** | | **400-m**  **walk time** | **HABCPPB** |
|  | | | | **Beta (95% CI) p-value** | | | | | |  |  |  |
| **Beta oxidation rate** | -0.041  (-0.115, 0.034)  0.282 | 0.038  (-0.032, 0.109)  0.285 | 0.053  (-0.018, 0.124)  0.145 | | 0.055  (-0.015, 0.125)  0.123 | 0.007  (-0.053, 0.066)  0.825 | 0.011  (-0.048, 0.071)  0.708 | -0.009  (-0.069, 0.052)  0.781 | 0.012  (-0.056, 0.079)  0.735 | | -0.037  (-0.106, 0.031)  0.287 | **-0.075**  **(-0.139, -0.010)**  **0.024** |
| **Acylcarnitines: long-chain** | 0.003  (-0.072, 0.078)  0.939 | 0.025  (-0.045, 0.095)  0.485 | 0.062  (-0.008, 0.132)  0.081 | | 0.046  (-0.023, 0.114)  0.190 | 0.034  (-0.025, 0.093)  0.257 | -0.025  (-0.084, 0.034)  0.404 | -0.016  (-0.075, 0.044)  0.601 | 0.058  (-0.009, 0.125)  0.090 | | 0.030  (-0.037, 0.097)  0.375 | 0.003  (-0.061, 0.068)  0.922 |
| **Acylcarnitines: medium-chain** | -0.009  (-0.083, 0.066)  0.818 | 0.035  (-0.038, 0.107)  0.346 | 0.069  (-0.003, 0.142)  0.059 | | **0.116**  **(0.047, 0.186)**  **0.001** | 0.048  (-0.013, 0.108)  0.123 | -0.043  (-0.103, 0.017)  0.161 | -0.004  (-0.065, 0.058)  0.910 | 0.026  (-0.041, 0.094)  0.449 | | -0.010  (-0.077, 0.057)  0.770 | 0.002  (-0.063, 0.067)  0.955 |
| **Acylcarnitines: short-chain** | -0.044  (-0.118, 0.031)  0.252 | 0.025  (-0.046, 0.096)  0.495 | 0.035  (-0.036, 0.107)  0.333 | | 0.012  (-0.058, 0.082)  0.742 | 0.009  (-0.051, 0.069)  0.762 | -0.017  (-0.077, 0.044)  0.589 | -0.020  (-0.081, 0.041)  0.514 | -0.017  (-0.085, 0.05)  0.614 | | -0.063  (-0.131, 0.006)  0.074 | **-0.082**  **(-0.147, -0.017)**  **0.014** |
| **Ceramides: very long-chain** | 0.015  (-0.063, 0.094)  0.699 | 0.057  (-0.018, 0.131)  0.134 | 0.047  (-0.028, 0.123)  0.216 | | 0.067  (-0.006, 0.140)  0.073 | 0.044  (-0.019, 0.107)  0.166 | 0.020  (-0.044, 0.083)  0.542 | -0.003  (-0.067, 0.061)  0.922 | **0.092**  **(0.021, 0.164)**  **0.011** | | **0.126**  **(0.054, 0.198)**  **0.001** | **0.096**  **(0.027, 0.165)**  **0.006** |
| **Ceramides: long-chain** | -0.039  (-0.115, 0.038)  0.323 | 0.026  (-0.047, 0.099)  0.483 | 0.051  (-0.024, 0.125)  0.183 | | **0.090**  **(0.018, 0.163)**  **0.014** | 0.034  (-0.029, 0.097)  0.290 | 0.014  (-0.049, 0.076)  0.669 | 0.015  (-0.049, 0.078)  0.651 | -0.004  (-0.074, 0.066)  0.914 | | 0.010  (-0.062, 0.081)  0.791 | -0.010  (-0.078, 0.057)  0.763 |
| **Glycosylceramides: very long-chain** | 0.014  (-0.061, 0.089)  0.712 | 0.064  (-0.007, 0.135)  0.076 | 0.053  (-0.018, 0.125)  0.144 | | **0.092**  **(0.022, 0.162)**  **0.010** | **0.065**  **(0.005, 0.125)**  **0.034** | 0.040  (-0.020, 0.101)  0.192 | 0.051  (-0.010, 0.112)  0.104 | **0.134**  **(0.067, 0.201)**  **<0.001** | | **0.164**  **(0.096, 0.232)**  **<0.001** | **0.173**  **(0.109, 0.237)**  **<0.001** |
| **Glycosylceramides: long-chain** | 0.025  (-0.052, 0.101)  0.525 | 0.063  (-0.009, 0.135)  0.088 | 0.053  (-0.020, 0.126)  0.154 | | **0.094**  **(0.022, 0.165)**  **0.010** | **0.075**  **(0.014, 0.136)**  **0.017** | 0.004  (-0.057, 0.066)  0.887 | 0.022  (-0.040, 0.085)  0.483 | 0.067  (-0.001, 0.136)  0.055 | | **0.105**  **(0.034, 0.175)**  **0.004** | **0.103**  **(0.037, 0.169)**  **0.002** |
| **Glycerolipids & cholesteryl esters: very long-chain** | 0.036  (-0.040, 0.111)  0.354 | 0.061  (-0.010, 0.132)  0.091 | 0.062  (-0.010, 0.134)  0.090 | | 0.062  (-0.008, 0.132)  0.082 | 0.041  (-0.019, 0.101)  0.181 | 0.028  (-0.032, 0.089)  0.359 | 0.038  (-0.023, 0.099)  0.224 | **0.137**  **(0.070, 0.204)**  **<0.001** | | **0.135**  **(0.067, 0.203)**  **<0.001** | **0.156**  **(0.092, 0.221)**  **<0.001** |
| **Glycerolipids & cholesteryl esters: long-chain** | 0.036  (-0.042, 0.114)  0.364 | 0.022  (-0.052, 0.096)  0.565 | 0.063  (-0.012, 0.138)  0.097 | | **0.074**  **(0.001, 0.147)**  **0.047** | 0.027  (-0.036, 0.090)  0.394 | 0.029  (-0.034, 0.092)  0.365 | 0.015  (-0.049, 0.079)  0.643 | **0.086**  **(0.015, 0.156)**  **0.017** | | **0.108**  **(0.038, 0.179)**  **0.003** | **0.116**  **(0.048, 0.183)**  **0.001** |
| **Sphingomyelins: very long-chain** | 0.072  (-0.004, 0.148)  0.065 | **0.111**  **(0.038, 0.183)**  **0.003** | 0.048  (-0.025, 0.122)  0.199 | | **0.086**  **(0.015, 0.157)**  **0.018** | 0.058  (-0.003, 0.119)  0.064 | **0.082**  **(0.020, 0.143)**  **0.009** | **0.085**  **(0.023, 0.147)**  **0.007** | **0.161**  **(0.093, 0.229)**  **<0.001** | | **0.216**  **(0.147, 0.285)**  **<0.001** | **0.189**  **(0.123, 0.255)**  **<0.001** |
| **Sphingomyelins: long-chain** | 0.029  (-0.052, 0.110)  0.477 | **0.095**  **(0.019, 0.172)**  **0.014** | 0.074  (-0.004, 0.152)  0.062 | | **0.124**  **(0.049, 0.200)**  **0.001** | **0.069**  **(0.004, 0.134)**  **0.038** | 0.062  (-0.003, 0.127)  0.062 | 0.053  (-0.013, 0.119)  0.115 | **0.078**  **(0.004, 0.151)**  **0.038** | | **0.111**  **(0.036, 0.185)**  **0.004** | **0.075**  **(0.004, 0.146)**  **0.039** |
| **Triacylglycerides: very long-chain** | -0.019  (-0.092, 0.054)  0.612 | -0.016  (-0.086, 0.053)  0.642 | 0.014  (-0.057, 0.084)  0.705 | | 0.003  (-0.066, 0.072)  0.928 | 0.012  (-0.047, 0.072)  0.683 | 0.040  (-0.018, 0.099)  0.179 | -0.014  (-0.073, 0.046)  0.651 | -0.050  (-0.117, 0.016)  0.140 | | -0.028  (-0.096, 0.039)  0.410 | -0.015  (-0.079, 0.050)  0.656 |
| **Triacylglycerides: long-chain** | -0.049  (-0.123, 0.024)  0.188 | -0.048  (-0.119, 0.022)  0.177 | -0.013  (-0.085, 0.058)  0.712 | | -0.007  (-0.076, 0.063)  0.847 | -0.036  (-0.097, 0.024)  0.234 | -0.002  (-0.061, 0.058)  0.952 | -0.048  (-0.108, 0.012)  0.117 | -0.064  (-0.131, 0.003)  0.060 | | -0.056  (-0.125, 0.012)  0.107 | -0.043  (-0.107, 0.022)  0.193 |

Note: Values of olfaction, cognition, and mobility measures are standardized to Z-scores. All models were adjusted for age, sex, and race and additionally adjusted for olfaction test version, education, and height for olfaction, cognition, and mobility outcomes, respectively. Z scores of TMT-A, TMT-B, and 400-meter walk time were flipped to be in consistent directions with other outcome measures. Bold number indicates significance at p<0.05. CVLT: California Verbal Learning Test; HABCPPB: Health Aging and Body Composition Physical Performance Battery.

**Supplementary Table 3. Associations of lipids with white matter integrity, diet, and visceral fat in subsamples.**

| **Fractional anisotropy (n=453)** | **Very long-chain glycosylceramides** | | **Long-chain glycosylceramides** | | **Very long-chain sphingomyelins** | | **Long-chain sphingomyelins** | |
| --- | --- | --- | --- | --- | --- | --- | --- | --- |
|  | **beta** | **p** | **beta** | **p** | **beta** | **p** | **beta** | **p** |
| Genu of corpus callosum | 0.008 | 0.241 | 0.014 | 0.186 | 0.030 | 0.051 | 0.017 | 0.281 |
| Body of corpus callosum | 0.011 | 0.130 | 0.020 | 0.074 | 0.033 | **0.043** | 0.027 | 0.102 |
| Splenium of corpus callosum | 0.012 | 0.119 | 0.023 | 0.069 | 0.044 | **0.019** | 0.043 | **0.025** |
| External capsule | 0.002 | 0.780 | -0.002 | 0.879 | 0.011 | 0.447 | 0.007 | 0.623 |
| Uncinate fasciculus | 0.014 | 0.103 | 0.027 | **0.045** | 0.023 | 0.253 | 0.035 | 0.084 |
| Superior longitudinal fasciculus | 0.013 | 0.119 | 0.011 | 0.401 | 0.015 | 0.425 | 0.021 | 0.283 |
| Inferior longitudinal fasciculus | 0.009 | 0.269 | 0.006 | 0.645 | 0.008 | 0.682 | 0.010 | 0.593 |
| Superior fronto-occipital fasciculus | 0.009 | 0.268 | 0.017 | 0.178 | 0.036 | 0.051 | 0.023 | 0.231 |
| Inferior fronto-occipital fasciculus | 0.015 | 0.055 | 0.011 | 0.383 | 0.022 | 0.220 | 0.003 | 0.857 |
| Cingulate part of the cingulum | 0.006 | 0.429 | 0.008 | 0.523 | 0.019 | 0.296 | 0.013 | 0.487 |
| Hippocampal part of the cingulum | 0.019 | **0.014** | 0.027 | **0.030** | 0.065 | **0.000** | 0.044 | **0.018** |
| Fornix (column and body) | 0.013 | **0.050** | 0.022 | **0.039** | 0.049 | **0.002** | 0.037 | **0.022** |
| Fornix (cres) Stria terminalis | 0.006 | 0.393 | 0.013 | 0.218 | 0.050 | **0.001** | 0.033 | **0.036** |
| Anterior limb of internal capsule | 0.011 | 0.129 | 0.016 | 0.182 | 0.034 | 0.051 | 0.022 | 0.227 |
| Posterior limb of internal capsule | -0.011 | 0.182 | -0.026 | 0.055 | -0.020 | 0.305 | -0.016 | 0.433 |
| Anterior corona radiata | 0.007 | 0.280 | 0.014 | 0.172 | 0.027 | 0.068 | 0.024 | 0.107 |
| Superior corona radiata | -0.005 | 0.558 | -0.014 | 0.274 | -0.018 | 0.361 | -0.026 | 0.184 |
| Posterior corona radiata | 0.000 | 0.993 | -0.009 | 0.520 | -0.016 | 0.426 | -0.014 | 0.509 |
| Cerebellar peduncle | 0.001 | 0.929 | -0.006 | 0.652 | 0.004 | 0.820 | 0.002 | 0.905 |
| **Diet^ (n=456)** | **beta** | **p** | **beta** | **p** | **beta** | **p** | **beta** | **p** |
| Alternate healthy eating index | 0.115 | 0.098 | 0.103 | 0.368 | 0.086 | 0.612 | -0.195 | 0.256 |
| Mediterranean-like diet | 0.032 | **0.015** | 0.015 | 0.496 | 0.049 | 0.127 | -0.025 | 0.452 |
| Mediterranean-like diet that is specifically for the brain | 0.030 | **0.010** | 0.015 | 0.423 | 0.042 | 0.135 | -0.015 | 0.587 |
| Total Protein, g | -0.103 | 0.426 | -0.203 | 0.339 | 0.290 | 0.357 | 0.473 | 0.139 |
| Vegetable Protein, g | 0.040 | 0.487 | -0.091 | 0.338 | -0.097 | 0.488 | -0.504 | **<0.001** |
| Animal Protein, g | -0.143 | 0.287 | -0.111 | 0.615 | 0.388 | 0.235 | 0.980 | **0.003** |
| Total Fat, g | -0.122 | 0.256 | -0.245 | 0.163 | 0.303 | 0.245 | 0.348 | 0.189 |
| Total Saturated Fatty Acids, g | -0.088 | 0.062 | -0.099 | 0.202 | 0.084 | 0.468 | 0.186 | 0.113 |
| Total Monounsaturated Fatty Acids, g | -0.039 | 0.372 | -0.103 | 0.148 | 0.115 | 0.278 | 0.120 | 0.266 |
| Total Polyunsaturated Fatty Acids, g | 0.016 | 0.674 | -0.016 | 0.794 | 0.072 | 0.430 | 0.005 | 0.954 |
| Total Trans-Fatty Acids, g | -0.013 | 0.416 | -0.017 | 0.520 | 0.007 | 0.863 | -0.022 | 0.594 |
| Omega-3 Fatty Acids, g | -0.002 | 0.541 | -0.003 | 0.625 | -0.006 | 0.492 | 0.002 | 0.808 |
| **Visceral fat (n=570)** | -243 | **<0.001** | -368 | **<0.001** | -366 | **<0.001** | -31 | 0.784 |

Note: ^ Models of diet were adjusted for total calorie intake. Bold indicates p<0.05.
